# Collaboration in orthodontic clinical trials: prevalence and association with sample size and funding

**DOI:** 10.1186/s40510-018-0215-3

**Published:** 2018-06-11

**Authors:** D. Al-Moghrabi, A. Tsichlaki, N. Pandis, P. S. Fleming

**Affiliations:** 1 Centre for Oral Growth and Development, Barts and The London School of Medicine and Dentistry, Queen Mary University of London, London, UK; 2 College of Dentistry, Princess Nourah bint Abdulrahman University, Riyadh, Saudi Arabia; 3 Department of Orthodontics, Dental School, Medical Faculty, University of Bern, Bern, Switzerland; 4 Corfu, Greece

## Abstract

**Background:**

To assess patterns of research collaboration in orthodontics and possible relationships with sample size and funding status.

**Methods:**

Orthodontic randomised and non-randomised controlled clinical trials published between 2013 and 2017 were identified through electronic searching. The nature of collaboration, author institutions, study setting, sample size, and funding status were assessed. Linear and logistic regression analyses were applied.

**Results:**

Of 1153 studies, 217 met the selection criteria. The majority of studies were authored by university academics (86%), were conducted in a single centre (71.9%) and in at least one university hospital (68.2%). The number of practice-based trials (10.1%), as well as the involvement of specialist practitioners (5.2%) in co-authorship, was limited. Multi-centred studies within a single country were associated with a significantly larger sample size compared to single-centred trials (*P* = 0.00; 95% confidence interval [CI] 33.59, 106.93). However, authorship collaboration either nationally (odds ratio [OR] 2.37; 95% CI 0.85, 6.57) or internationally across different continents (OR 5.54; 95% CI 0.62, 49.52) did not translate into increased funding.

**Conclusions:**

Most orthodontic studies were undertaken in university hospital settings within a single country. Collaboration is common in orthodontics but involvement of practice settings remains limited, suggesting a need for stimulation of practice-based research and research partnerships.

**Electronic supplementary material:**

The online version of this article (10.1186/s40510-018-0215-3) contains supplementary material, which is available to authorized users.

## Background

Collaborative research facilitates dissemination of knowledge and sharing of skills between researchers and, when performed optimally, may also promote holistic and relevant research outputs. Collaboration can also help in expediting the conduct of research, and may be beneficial in minimising associated costs, especially in studies with specific technical requirements [1]. Previous literature has suggested that collaborative research may translate into higher research quality, citation counts and may attract more funding than non-collaborative research [2–4].

A research partnership can be formed at different levels: local (within the same institution), national (different institutions within the same country) or international [5]. International collaboration, in particular, has been positively correlated with citation counts in basic science journals [3, 6]. Within biomedical fields, research collaboration at national or international level can go beyond authorship level and can involve multi-centred trials and, subsequently, attainment of larger and more diverse samples. This is particularly important, as single-centred trials may carry the risk of overestimating effect sizes [7], thus reducing their external validity in comparison to multi-centred trials.

There is increasing recognition of the importance of bridging the translational gap between research and clinical practice in order to reduce research waste and improve patient care [8–10]. For instance, randomised controlled trials of coronary bypass surgery have been shown to be relevant to less than 15% of patients [11]. In terms of dental research, over a 3-year period, up to 44% of outcomes were primarily clinician-centred rather than patient-centred [12]. Furthermore, although clinical trials are essential to test the effectiveness of interventions in experimental and academic settings, practice-based research is a prerequisite to proving efficacy in day-to-day, ‘real-word’ clinical practice scenarios [10]. Therefore, there is a pressing need for research collaboration between academics and clinicians to ensure more relevant research questions and study designs are framed, with findings made applicable to clinical practice [13].

In the orthodontic literature, there are no studies indicating the status and characteristics of research partnerships. Therefore, the aims of this paper are to describe the prevalence and patterns of collaborative and non-collaborative research in orthodontics, and to evaluate the possible relationship between research collaboration and study sample size and funding status.

## Methods

The following electronic databases were searched by two authors (DA, AT) over a 5-year period from January 1st 2013 to December 31st 2017: Embase®, MEDLINE via Ovid, psycINFO via EBSCO and the Cochrane Central Register of Controlled Trials (CENTRAL) via the Cochrane Library with no language restrictions using specific search terms (Table 1). In addition, the reference lists of relevant studies were cross-checked for any unidentified relevant studies.

**Table 1:** Search strategy

Embase® search query	
1 orthodontic$ (75,005)	
2 #1 AND (‘clinical trial’/de OR ‘controlled clinical trial’/de OR ‘prospective study’/de OR ‘randomized controlled trial’/de) AND ‘article’/it (3360)	
3 #1 AND (‘clinical trial’/de OR ‘controlled clinical trial’/de OR ‘prospective study’/de OR ‘randomized controlled trial’/de) AND ‘article’/it AND ‘human’/de AND (2013:py OR 2014:py OR 2015:py OR 2016:py OR 2017:py) (1008)	

The following selection criteria were used to identify relevant studies:

- Study design: randomised controlled trials and controlled clinical trials
- Participants: orthodontic patients of any age group
- Intervention: any intervention
- Comparison: any comparison or control group
- Outcome: any outcome measure

All retrospective or pilot studies, review articles and systematic reviews were excluded. Studies involving patients undergoing orthognathic surgery, syndromic conditions, cleft lip, and/or palate or obstructive sleep apnoea were also omitted. Where multiple publications were derived from the sample, only one publication was randomly selected.

The abstracts of identified studies were assessed by two authors (DA, AT), and the full-texts of abstracts meeting the selection criteria were subsequently retrieved. Data were extracted using pre-piloted data collection forms by two authors (DA, AT). The following details were extracted from each study: (1) study design (randomised controlled trial or controlled clinical trial); (2) sample size; (3) number of authors, country and continent of authorship (Asia, Africa, Europe, Australia, and North and South America); (4) author discipline; and (5) number and type of setting (with studies classified as based in practice, university hospital, non-university hospital, or community). Where individual authors listed two or more institutional affiliations, only the first was recorded. The study was recorded as funded when an external funder was listed, or when study material was provided by manufacturers or industry. The source of funding was classified as university or research institution, non-profit organisation, professional society, practice, council, industry, government or multiple, where more than one of the previous applied.

Two researchers (DA, AT) classified the presence and nature of collaboration with disagreements resolved by joint discussion with a third author (PSF) as follows:

- Authorship level: based on the institutions listed in the affiliation being sub-classified into local (with all authors from the same institution), national (authors from different institutions but within the same country), and international (authors from different countries).
- Study conduct level: based on the setting, these were classified as single- or multi-centred studies. Furthermore, multi-centred studies were sub-classified into national (conducted in different institutions but in the same country) or international (conducted in different countries).

### Statistical analysis

Descriptive statistics were undertaken to calculate frequencies of categorical data. Linear and logistic regression analyses were applied to assess the possible association of collaboration with both the sample size and funding status, respectively. The level of statistical significance in all analyses was set at *P* = 0.05 with statistical analyses undertaken using Stata statistical software package (version 14.1; StataCorp, College Station, TX).

## Results

**Fig. 1:**
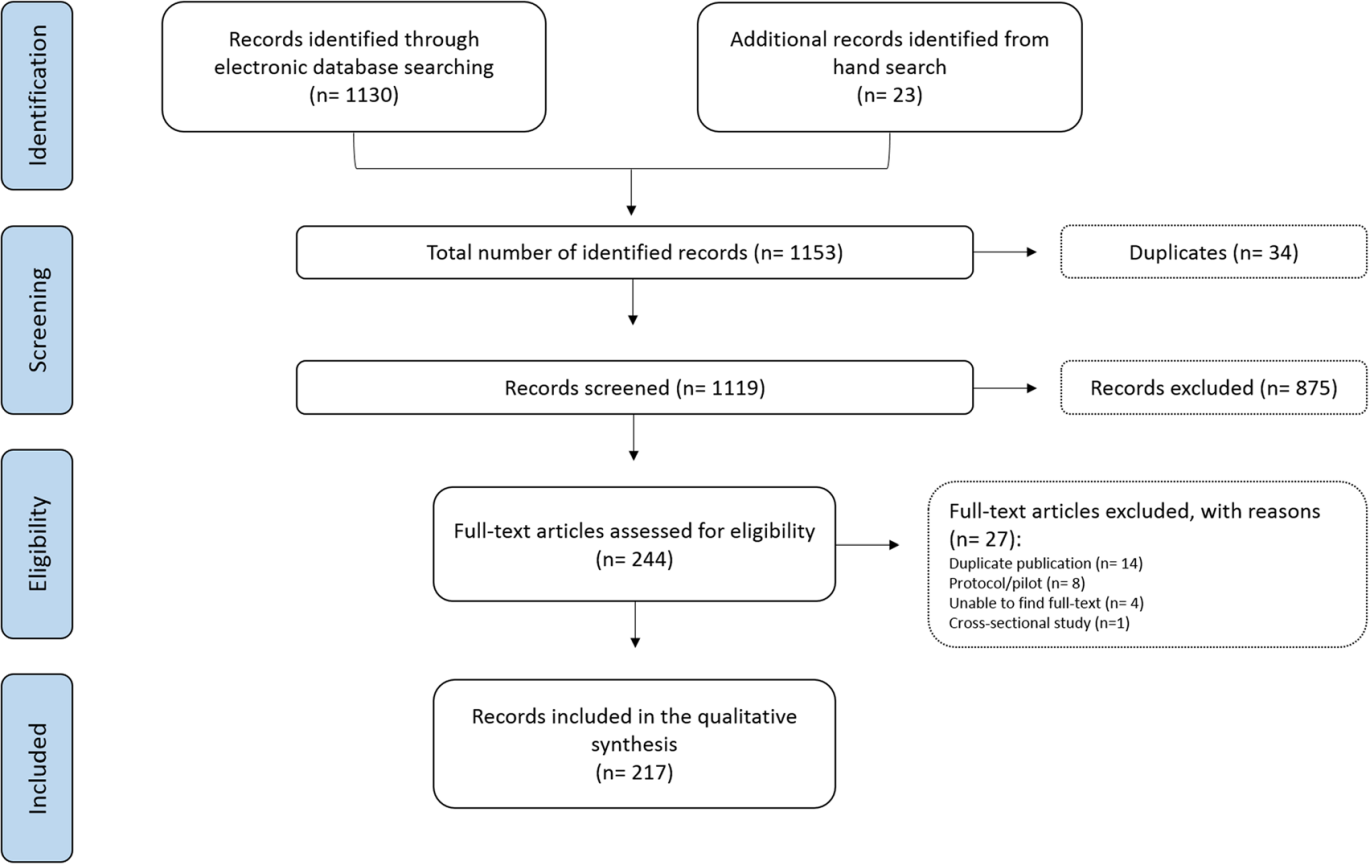
PRISMA flowchart of included studies

A total of 1153 studies were identified through electronic databases and reference lists of relevant studies. Only 244 were considered potentially relevant. Following retrieval of full-text articles, 217 (ranging from 41 to 45 per annum) studies met the inclusion criteria (Fig. 1). Descriptive data from each study are presented in the Additional file 1.

The mean number of authors per paper was 5 ± 2.01 authors. Approximately two-thirds of the included studies were co-authored by 3 to 6 authors (*n* = 160). The authors in the majority of the studies were based in a university or a university hospital (86%), while only 5.17% were based in practice (Table 2). The first author was an orthodontist in 151 studies. The rest of the authors included a minimum of one orthodontist, statistician or periodontist in 172, 31 and 18 studies, respectively.

**Table 2:** Authorship characteristics of included studies (*n* = 217)

Number of authors in each study	Number of studies
1–2	*n* = 15
3–6	*n* = 160
7–9	*n* = 37
> 9	*n* = 5
Institutions of authors	Number of authors
Community-based	*n* = 4
Non-university hospital	*n* = 61
University hospital	*n* = 930
Practice-based	*n* = 56
Other	*n* = 5
No information	*n* = 26

Thirty percent (*n* = 65) of the studies were limited to authors from the same institution. However, in most studies (*n* = 101; 46.5%), authors were from different institutions but within the same country. National authorship collaboration was particularly common among Brazilian (*n* = 13) and British (*n* = 12) centres. International authorship collaboration was identified in 44 studies (20.3%) studies; of these, 26 were undertaken in different continents. Studies involving European centres had the highest preponderance of international authorship collaboration overall (*n* = 24). However, Asian research groups were involved in the highest number of inter-continental authorship (*n* = 19), mainly working with researchers in North America (*n* = 9) and Europe (*n* = 4).

**Fig. 2:**
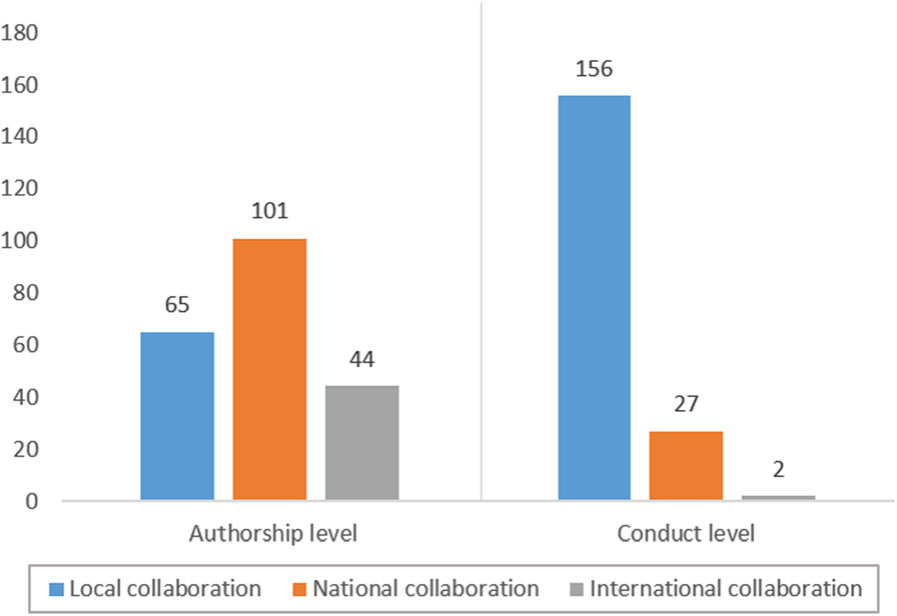
Number of studies conducted in at least one of the listed study settings. *19 studies were multi-centred being conducted in two different settings

**Fig. 3:**
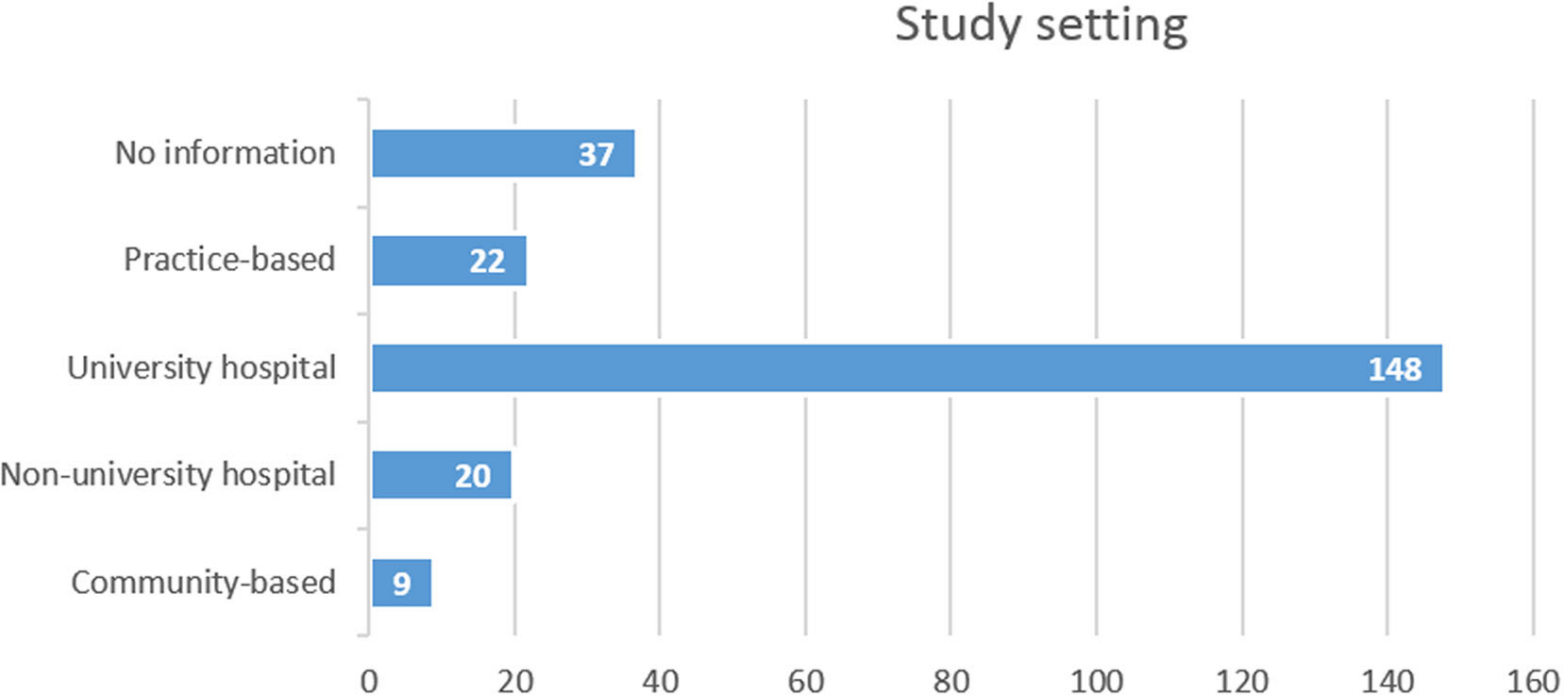
Distribution of studies within each level of collaboration

The number of studies conducted in at least one university hospital was 148 with only 22 trials being practice-based (Fig. 2). More than two-thirds of the trials were single-centred (*n* = 156), with only 29 being multi-centred. The majority of the multi-centred studies were conducted nationally (*n* = 27). The largest number of centres in one study was 12 general dental practices ; however, the majority of multi-centred studies were conducted in 2 to 3 centres (*n* = 22). Level of authorship collaboration does not necessarily translate into similar levels of conduct collaboration (Fig. 3). Although a high proportion of studies involved national or international co-authorship (*n* = 145), only 18.6% of these were multi-centred (*n* = 27). Similarly, out of 44 internationally co-authored studies (20.27%), only 4.55% (n = 2) were conducted internationally [14, 15].

Multi-centred studies within a single country were associated with a significantly larger sample size compared to single-centred trials (*P* < 0.01, 95% confidence interval [CI]: 33.59, 106.93; Table 3). However, international collaboration was not associated with the study sample size (*P* > 0.05) either at authorship or study conduct level.

**Table 3:** Results of linear and logistic regression analyses

	Sample size	Coefficient	Odds ratio	*P* value	95% CI
Sample size	Authorship collaboration				
	National collaboration	− 4.89	–	0.75	− 34.61, 24.84
	International collaboration within the same continent	14.54	–	0.56	− 34.47, 63.55
	International collaboration within different continents	0.17	–	0.1	− 34.93, 44.27
	Study conduct collaboration				
	National collaboration	70.26	–	0.00*	33.59, 106.93
	International collaboration within the same continent	89.58	–	0.31	− 84.09, 263.25
	International collaboration within different continents	− 30.42	–	0.73	− 204.09, 143.25
Funding status	Authorship collaboration				
	National collaboration	–	2.37	0.1	0.85, 6.57
	International collaboration within the same continent	–	1.00	–	–
	International collaboration within different continents	–	5.54	0.13	0.62, 49.52
	Study conduct collaboration	–	0.77	0.7	0.21, 3.43

*CI* confidence interval

*P <0.05

Eighty-two studies (37.79%) reported receiving funding or financial support; of those, only 15 were multi-centred. Most funded studies were conducted in at least one university hospital (*n* = 62). The source of funding in the majority of studies was from universities or research institutions (*n* = 25; 30.49%) followed by industry (*n* = 19; 23.17%). Twelve studies reported receiving funding from multiple sources. A limited number of studies received funding from professional societies (*n* = 9; 10.98%) or non-profit organisations (*n* = 8; 9.76%). A high proportion of studies conducted in Sweden (90%; *n* = 9), China (58.82%; *n* = 10) and the UK (40.91%; *n* = 9) were successful in obtaining funding.

Receipt of funding was slightly more prevalent in studies which involved national (OR 2.37; 95% CI 0.85, 6.57) or international authorship collaboration across different continents (OR 5.54; 95% CI 0.62, 49.52) in comparison to local collaboration; however, the differences were not statistically significant (Table 3).

## Discussion

The majority of published trials in orthodontics over a 5-year period were co-authored nationally by academics, conducted in at least one university hospital and were single-centred. Practice-based trials and the involvement of specialist practitioners in co-authorship were comparatively rare. Larger samples were characteristic of multi-centred studies within a single country in comparison to single-centred studies. Although the majority of identified studies were authored by orthodontists, inter-disciplinary collaboration was found most frequently with periodontists in 14.3% of the identified studies. Based on the present findings, research collaboration in orthodontics does not seem to attract more funding. This differs from collaborative research in implantology which was 1.5 times more likely to be funded compared to non-collaborative [16]. However, other factors such as the geographic region and study type had an impact on funding status in implantology [17].

The preponderance of orthodontic studies being conducted in university hospitals highlighted in the present research reflects findings from a recent orthodontic systematic review [18], comprising meta-analysis of 22 studies, all of which were exclusively undertaken in university and/or hospital settings. Although many academic orthodontists were affiliated to practice as well as university hospitals, the tendency to conduct studies in the latter was clear. The receipt of funding from university or research institution in many instances may contribute to this (30.49%). Furthermore, the centrality of research outputs to academic recognition and promotion creates a much more compelling incentive to undertake research among academics than practice-based clinicians [19, 20].

In some countries, such as the UK, orthodontic treatment is often carried out in an academic setting and funded through national healthcare systems, and may only be provided to children and young people with severe malocclusion [21]. Patients treated in different settings may not share similar characteristics, therefore, limiting the possible applicability of findings from specific settings to other areas. As such, clear reporting of the study setting is important within any trial and is also stipulated within the Consolidated Standards of Reporting Trials statement [22]. Notwithstanding this, 17.1% of the identified studies did not report the study setting. Suboptimal reporting of study setting has previously been highlighted in 27.3% orthodontic randomised controlled trials published between 2001 and 2013 [23].

There has been a recognition of the importance of orthodontic practice-based research [24]. This can be fostered by encouraging clinicians to engage with academics working closely throughout the process. This is especially important as clinicians might be reluctant to partake in research due to limitations in terms of time and funding [25], allied to the logistical implications of research in practice. Depending on the research question posed, practice-based research can be undertaken in the form of either randomised controlled trials or other ideally prospective designs, as randomisation may be more complicated to undertake in a private healthcare setting. The limited involvement of specialist practitioners in research has been identified in the medical field, with only 9% of general practitioners having published in peer-reviewed journals [25]. Academic researchers might be more familiar with setting up a clinical study, synthesising information and applying for funding; however, specialist practitioners can pose relevant research questions and tailor research to realistic practice-based scenarios making the argument for pooling of resources compelling. The involvement of specialist practitioners in research may be rewarding, can form part of professional development and can assist in raising the profile and reputation of a practice. As such, it is important that engagement of academics with potential practice-based researchers is viewed as mutually beneficial. Positive strides have been made in terms of imbedding collaboration with practice-based researchers in recent years with the establishment and funding of the National Dental Practice-Based Research Network in the USA supported by the National Institute of Dental and Craniofacial Research leading to clinical research evaluating the management of white spot lesions [26] and ongoing research evaluating the stability of open bite correction.

There has been increased acknowledgement of the importance of big data research in healthcare [27]. This has been possible with the advances in technology such as the use of electronic health records and centralised online databases allowing information sharing and safe data transfer. Inherent challenges to big data research may be addressed by setting up practice-based collaborative research. Calls have recently been made for the establishment of large practice-based databases to allow evaluation of the predictability of orthodontic treatments potentially making the use of big data in orthodontics less aspirational [28].

This is the first study describing collaboration patterns within the orthodontic literature. A distinction was made between authorship and conduct collaboration, as research can be authored by detached researchers with remote involvement and expertise, while recruitment and conduct actually only occurred in one site. This was clearly evident in the present sample in light of the discrepancy highlighted, with collaboration far less common at study conduct level. Only randomised controlled trials and controlled clinical trials were included in the present study; as such, the observed patterns may not apply to observational designs. The sample included in this study was limited to published trials over the most recent 5-year period with no language or journal of publication restrictions; as such, it is likely to be representative of contemporary orthodontic trials.

## Conclusions

Orthodontic trials are most often conducted in academic settings by university-based authors. There is a need to improve engagement with research in primary care and among specialist practitioners in order to stimulate and produce research that is most applicable to the majority of orthodontic patients.

## Additional file

Additional file 1: Characteristics of the included studies (*n* = 217). (DOCX 58 kb)
