# Supplementary material for: Collaboration in orthodontic clinical trials: prevalence and association with sample size and funding
Source: Prog Orthod. 2018 Jun 11;19:16. doi: 10.1186/s40510-018-0215-3 (PMC5994391; doi:10.1186/s40510-018-0215-3)
Supplement: Supplementary file 1 — Characteristics of the included studies (n = 217). (DOCX 58 kb) [file 40510_2018_215_MOESM1_ESM.docx]

Additional file 1. Characteristics of the included studies (n= 217)

| **Study** | **Year of publication** | **Sample size** | **Funding status** | **Single-centred or multi-centred** | **Study setting** | **Authorship collaboration** | **Study conduct collaboration** |
| --- | --- | --- | --- | --- | --- | --- | --- |
|  |  |  |  |  | 1. university hospital  2: non-university hospital  3: practice-based  4: community-based | 1: local collaboration  2: national collaboration  3: international collaboration (same continent)  4: international collaboration (different continents) | |
| Albanna | 2017 | 82 | yes | single | 1 | 2 | 1 |
| Alkadhi | 2017 | 44 | NI | single | 1 | 1 | 1 |
| AlSayed Hasan | 2017 | 26 | no | single | 1 | 1 | 1 |
| AlSayed Hasan | 2017 | 26 | no | single | 1 | 1 | 1 |
| Andrucioli | 2017 | 29 | NI | NI | NI | 2 | NI |
| Atik | 2017 | 30 | NI | single | 1 | 1 | 1 |
| Bazargani | 2017 | 40 | yes | single | 1 | 3 | 1 |
| Beerens | 2017 | 65 | no | single | 1 | 1 | 1 |
| Bock | 2017 | 46 | yes | single | 1 | 2 | 1 |
| Canan | 2017 | 53 | yes | single | 1 | 2 | 1 |
| Durrani | 2017 | 60 | NI | single | 1 | 2 | 1 |
| Eissa | 2017 | 45 | NI | single | NI | 4 | 1 |
| Eslamian | 2017 | 41 | no | multi | 1, 3 | 2 | 2 |
| Forde | 2017 | 60 | NI | multi | 1,2 | 2 | 2 |
| Garry | 2017 | 12 | yes | single | 1 | 1 | 1 |
| Hemmatpour | 2017 | 45 | no | NI | NI | 1 | NI |
| Herrera | 2017 | 63 | yes | single | 1 | 1 | 1 |
| Jahanbin | 2017 | 25 | NI | single | 1 | 2 | 1 |
| Julku | 2017 | 67 | yes | multi | 4 | 1 | 2 |
| Jung | 2017 | 33 | NI | single | 1 | 4 | 1 |
| Kaklamanos | 2017 | 22 | yes | single | 1 | 2 | 1 |
| Kaur | 2017 | 25 | no | single | 1 | 1 | 1 |
| Lin | 2017 | 160 | yes | single | 1 | 4 | 1 |
| Lione | 2017 | 42 | NI | single | 1 | 3 | 1 |
| Lione | 2017 | 30 | NI | single | 1 | 4 | 1 |
| Little | 2017 | 120 | NI | single | 2 | 1 | 1 |
| Maurya | 2017 | 45 | NI | NI | NI | 2 | NI |
| Miamoto | 2017 | 30 | NI | single | 1 | 2 | 1 |
| Nahas | 2017 | 40 | yes | single | 1 | 2 | 1 |
| Parker | 2017 | 72 | NI | single | 1 | 1 | 1 |
| Patterson | 2017 | 14 | yes | single | 1 | 2 | 1 |
| Penning | 2017 | 180 | yes | multi | 3 | 2 | 2 |
| Raghavan | 2017 | 45 | NI | single | 1 | 1 | 1 |
| Saleh | 2017 | 94 | yes | single | 3 | 4 | 1 |
| Samantha | 2017 | 33 | no | NI | NI | 1 | NI |
| Schatzle | 2017 | 35 | yes | single | 1 | 3 | 1 |
| Shah | 2017 | 22 | yes | single | 1 | 2 | 1 |
| Slaviero | 2017 | 50 | NI | NI | NI | 2 | NI |
| Ulhaq | 2017 | 150 | NI | multi | 1,2 | 4 | 3 |
| Ureturk | 2017 | 15 | yes | single | 1 | 1 | 1 |
| Varga | 2017 | 176 | NI | single | 1 | 2 | 1 |
| Wan | 2017 | 20 | yes | single | NI | 2 | 1 |
| White | 2017 | 41 | yes | single | 1 | 2 | 1 |
| Zhang | 2017 | 169 | NI | single | 2 | 1 | 1 |
| Zhu | 2017 | 80 | NI | single | 1 | 2 | 1 |
| Abbas | 2016 | 20 | NI | NI | NI | 1 | NI |
| Aglarci | 2016 | 59 | NI | NI | NI | 2 | NI |
| Almallah | 2016 | 36 | no | single | 1 | 1 | 1 |
| Atik | 2016 | 46 | NI | single | 1 | 1 | 1 |
| Bayani | 2016 | 100 | NI | single | 1 | 2 | 1 |
| Baysal | 2016 | 36 | yes | single | 1 | 1 | 1 |
| Bazargani | 2016 | 49 | NI | single | 1 | 2 | 1 |
| Cirgic | 2016 | 97 | yes | multi | NI | 2 | 2 |
| Cozzani | 2016 | 84 | NI | single | 2 | 4 | 1 |
| DiBiase | 2016 | 81 | yes | multi | 1, 2 | 3 | 2 |
| Elnagar | 2016 | 30 | NI | multi | 1 | 4 | 4 |
| Eslamian | 2016 | 30 | NI | single | 1 | 4 | 1 |
| Eslamian | 2016 | 20 | NI | NI | NI | 4 | NI |
| Farhadian | 2016 | 66 | yes | single | 1 | 1 | 1 |
| Farias | 2016 | 30 | NI | single | 3 | 1 | 1 |
| Ganzer | 2016 | 80 | yes | single | 4 | 2 | 1 |
| Garcia | 2016 | 41 | NI | single | 1 | 2 | 1 |
| Halicioglu | 2016 | 32 | NI | single | 1 | 2 | 1 |
| He | 2016 | 240 | yes | single | 1 | 1 | 1 |
| Hennessy | 2016 | 44 | yes | single | 1 | 2 | 1 |
| Ireland | 2016 | 1000 | yes | multi | 2 | 2 | 2 |
| Jia | 2016 | 85 | NI | multi | 1 | 2 | 2 |
| Lamberton | 2016 | 24 | yes | single | 1 | 2 | 1 |
| Leite | 2016 | 45 | yes | single | 1 | 2 | 1 |
| Lione | 2016 | 50 | NI | multi | 1 | 2 | 2 |
| Lobre | 2016 | 58 | NI | NI | NI | 2 | NI |
| Mandall | 2016 | 73 | yes | multi | 1, 2 | 2 | 2 |
| Martin | 2016 | 30 | NI | single | 1 | 2 | 1 |
| Masjedi | 2016 | 46 | no | single | 1 | 2 | 1 |
| Masjedi | 2016 | 42 | no | single | 1 | 2 | 1 |
| Maspero | 2016 | 20 | NI | single | 1 | 1 | 1 |
| Migliorati | 2016 | 52 | NI | single | 3 | 2 | 1 |
| Miles | 2016 | 40 | yes | single | 3 | 2 | 1 |
| Mohammed | 2016 | 22 | NI | single | 1 | 4 | 1 |
| Nahas | 2016 | 40 | yes | single | 1 | 2 | 1 |
| O'Dywer | 2016 | 138 | NI | multi | 1, 2 | 3 | 2 |
| O'Rourke | 2016 | 82 | no | single | 1 | 1 | 1 |
| Ozkan | 2016 | 36 | NI | NI | NI | 2 | NI |
| Perrini | 2016 | 24 | NI | single | 3 | 2 | 1 |
| Qamruddin | 2016 | 88 | NI | single | 1 | 3 | 1 |
| Rennick | 2016 | 46 | yes | single | 1 | 2 | 1 |
| Singh | 2016 | 45 | NI | NI | NI | 2 | NI |
| Yassaei | 2016 | 11 | yes | single | 1 | 2 | 1 |
| Abbate | 2015 | 50 | NI | single | 1 | 3 | 1 |
| Abdelrahman | 2015 | 87 | NI | multi | 1, 3 | 3 | 2 |
| Al-Anezi | 2015 | 24 | NI | NI | NI | NI | NI |
| Al-Silwadi | 2015 | 67 | no | single | 1 | 1 | 1 |
| Amini | 2015 | 30 | no | single | 1 | 1 | 1 |
| Ashkenazi | 2015 | 27 | no | multi | 1, 3 | 4 | 2 |
| Burhan | 2015 | 44 | no | single | 1 | 2 | 1 |
| Canturk | 2015 | 36 | NI | NI | NI | 2 | NI |
| Cardoso | 2015 | 16 | yes | single | 1 | 2 | 1 |
| Chung | 2015 | 11 | yes | single | 1 | 1 | 1 |
| de Castro Serafim | 2015 | 22 | NI | NI | NI | 2 | NI |
| Edman Tynelius | 2015 | 75 | yes | single | 4 | 1 | 1 |
| Elkordy | 2015 | 32 | no | single | 1 | 1 | 1 |
| Farhadian | 2015 | 72 | yes | single | 1 | 1 | 1 |
| Faria Junior | 2015 | 10 | NI | NI | NI | 2 | NI |
| Goncalves Zenóbio | 2015 | 13 | NI | single | 1 | 2 | 1 |
| Guler | 2015 | 30 | NI | single | 1 | 2 | 1 |
| Hofman | 2015 | 48 | NI | single | 1 | NI | 1 |
| Ileri | 2015 | 38 | NI | single | 1 | 1 | 1 |
| Jena | 2015 | 40 | yes | NI | NI | 2 | NI |
| Jiang | 2015 | 18 | NI | NI | NI | 4 | NI |
| Kaygisiz | 2015 | 60 | NI | single | 1 | 2 | 1 |
| Koopman | 2015 | 120 | yes | single | 1 | 1 | 1 |
| Lin | 2015 | 326 | NI | NI | NI | 1 | NI |
| Liu | 2015 | 44 | yes | single | 1 | 1 | 1 |
| Myrlund | 2015 | 48 | yes | multi | 1, 4 | 3 | 2 |
| Najafi | 2015 | 321 | yes | single | 1 | 4 | 1 |
| Nandhra | 2015 | 92 | no | multi | 1, 2 | 2 | 2 |
| Naoumova | 2015 | 67 | yes | single | 1 | 1 | 1 |
| Parkin | 2015 | 81 | yes | multi | 1, 2 | 2 | 2 |
| Paschoal | 2015 | 45 | yes | single | 3 | NI | 1 |
| Pawlak | 2015 | 70 | yes | single | 1 | 2 | 1 |
| Pires | 2015 | 35 | NI | single | 3 | 4 | 1 |
| Pithon | 2015 | 70 | NI | single | 3 | 2 | 1 |
| Qudeimat | 2015 | 36 | NI | single | 2 | 4 | 1 |
| Saffari | 2015 | 30 | NI | single | 1 | 2 | 1 |
| Sarul | 2015 | 270 | yes | single | 1 | 1 | 1 |
| Showkatbakhsh | 2015 | 60 | NI | NI | NI | 4 | NI |
| Sobouti | 2015 | 30 | no | single | 3 | 2 | 1 |
| Ugolini | 2015 | 70 | NI | multi | 1 | 2 | 2 |
| van der Kaaij | 2015 | 81 | NI | single | 1 | 2 | 1 |
| Wang | 2015 | 24 | yes | single | 1 | 2 | 1 |
| Wiedel | 2015 | 62 | yes | multi | 1, 4 | 2 | 2 |
| Woodhouse | 2015 | 81 | yes | multi | 1, 2 | 2 | 2 |
| Al-Naoum | 2014 | 30 | yes | single | 1 | 3 | 1 |
| Al-Sibaie | 2014 | 56 | yes | single | 1 | 2 | 1 |
| Alzahawi | 2014 | 82 | NI | single | 3 | 3 | 1 |
| Atwa | 2014 | 20 | no | NI | NI | 3 | NI |
| Baysal | 2014 | 67 | yes | single | 1 | 1 | 1 |
| Bazargani | 2014 | 24 | yes | single | 1 | 1 | 1 |
| Bovali | 2014 | 64 | no | single | 1 | 1 | 1 |
| Bradley | 2014 | 61 | NI | NI | NI | 4 | NI |
| Celar | 2014 | 21 | NI | single | 1 | 4 | 1 |
| da Costa Monini | 2014 | 25 | NI | single | 1 | 1 | 1 |
| Eppright | 2014 | 42 | NI | single | 1 | 1 | 1 |
| Eslamian | 2014 | 37 | yes | single | 1 | 4 | 1 |
| Falkensammer | 2014 | 26 | NI | single | 1 | 2 | 1 |
| Ferrari | 2014 | 32 | NI | NI | NI | NI | NI |
| Gupta | 2014 | 45 | NI | single | 1 | 2 | 1 |
| Heshmat | 2014 | 30 | NI | single | 3 | NI | 1 |
| Ireland | 2014 | 24 | yes | single | 1 | 1 | 1 |
| Kansal | 2014 | 10 | NI | single | 1 | 2 | 1 |
| Khattab | 2014 | 58 | yes | single | 1 | 2 | 1 |
| Lee | 2014 | 150 | NI | multi | 1, 2 | 2 | 2 |
| Lin | 2014 | 45 | NI | single | 1 | 1 | 1 |
| Mai | 2014 | 60 | NI | single | 1 | NI | 1 |
| Marini | 2014 | 60 | NI | single | 1 | 2 | 1 |
| Mazzoleni | 2014 | 40 | NI | single | 1 | 1 | 1 |
| Miresmaeili | 2014 | 20 | yes | NI | NI | 2 | NI |
| Nalcaci | 2014 | 46 | NI | single | 1 | 2 | 1 |
| Ozer | 2014 | 57 | NI | NI | NI | 2 | NI |
| Peng | 2014 | 148 | yes | single | 1 | 4 | 1 |
| Pinto | 2014 | 30 | yes | single | 1 | 2 | 1 |
| Rai | 2014 | 24 | no | single | 1 | 1 | 1 |
| Sandler | 2014 | 78 | yes | multi | 2 | 2 | 2 |
| Santamaria | 2014 | 34 | NI | NI | NI | 1 | NI |
| Son | 2014 | 70 | NI | single | 1 | 1 | 1 |
| Sonesson | 2014 | 424 | yes | multi | 1, 4 | 3 | 2 |
| Songra | 2014 | 100 | no | single | 2 | 2 | 1 |
| Songsiripradubboon | 2014 | 14 | yes | single | NI | 3 | 1 |
| Sudakhar | 2014 | 154 | no | single | 1 | 2 | 1 |
| Thakare | 2014 | 40 | yes | single | 1 | 1 | 1 |
| Torkan | 2014 | 40 | no | single | 1 | 2 | 1 |
| Tuncer | 2014 | 48 | yes | NI | NI | 2 | NI |
| Venancio | 2014 | 60 | yes | single | 1 | 2 | 1 |
| Vijayakumar | 2014 | 32 | NI | NI | NI | 1 | NI |
| Wang | 2014 | 60 | yes | single | 1 | 2 | 1 |
| Zingler | 2014 | 126 | yes | single | 3 | 3 | 1 |
| Al Maaitah | 2013 | 38 | NI | single | 1 | 1 | 1 |
| Baygin | 2013 | 60 | NI | single | 1 | 1 | 1 |
| Bechtold | 2013 | 40 | NI | single | 1 | 2 | 1 |
| Brunetto | 2013 | 59 | no | single | 1 | 2 | 1 |
| Canavarro | 2013 | 16 | yes | single | 1 | 4 | 1 |
| Cattaneo | 2013 | 64 | NI | single | 1 | 2 | 1 |
| Chen | 2013 | 90 | yes | single | 1 | 1 | 1 |
| Dalessandri | 2013 | 14 | NI | single | 3 | 2 | 1 |
| Dominguez | 2013 | 60 | no | single | 3 | 1 | 1 |
| Erbe | 2013 | 46 | yes | single | 1 | 4 | 1 |
| Eslamian | 2013 | 30 | yes | multi | 1, 3 | 4 | 2 |
| Fleming | 2013 | 101 | no | multi | 1, 2 | 1 | 2 |
| Gravina | 2013 | 36 | no | NI | NI | 2 | NI |
| Huang | 2013 | 135 | yes | multi | 3 | 2 | 2 |
| Isik Aslan | 2013 | 16 | NI | NI | NI | 1 | NI |
| Jiang | 2013 | 100 | yes | single | 1 | 1 | 1 |
| Jose | 2013 | 60 | NI | single | 1 | 1 | 1 |
| Keith | 2013 | 39 | NI | NI | NI | 1 | 1 |
| Kim | 2013 | 88 | NI | single | 3 | 4 | 1 |
| Knosel | 2013 | 21 | yes | single | 1 | 1 | 1 |
| Lagravere | 2013 | 62 | NI | single | 1 | 1 | 1 |
| Lombardo | 2013 | 20 | NI | NI | NI | 3 | NI |
| Mangnall | 2013 | 90 | NI | multi | 1, 2 | 2 | 2 |
| Marini | 2013 | 120 | NI | single | 1 | 2 | 1 |
| Martina | 2013 | 46 | yes | single | 1 | 4 | 1 |
| Nobrega | 2013 | 60 | yes | single | 4 | 2 | 1 |
| Pandis | 2013 | 220 | NI | single | 3 | 3 | 1 |
| Parkin | 2013 | 81 | yes | multi | 1, 2 | 2 | 2 |
| Pejda | 2013 | 38 | NI | single | 1 | 2 | 1 |
| Saleh | 2013 | 72 | yes | single | 1 | 1 | 1 |
| Salehi | 2013 | 156 | NI | single | 1 | 1 | 1 |
| Sandhu | 2013 | 96 | NI | single | NI | 2 | 1 |
| Senestrato | 2013 | 30 | yes | single | 1 | 2 | 1 |
| Shetty | 2013 | 42 | NI | NI | NI | 2 | NI |
| Showkatbakhsh | 2013 | 50 | NI | single | 1 | NI | 1 |
| Skold-Larsson | 2013 | 64 | yes | single | 4 | 2 | 1 |
| Smailiene | 2013 | 43 | NI | single | 1 | 3 | 1 |
| Srai | 2013 | 90 | NI | single | 1 | 1 | 1 |
| To | 2013 | 30 | yes | single | 4 | 1 | NI |
| Wong | 2013 | 45 | NI | single | 2 | 2 | 1 |
| Xu | 2013 | 165 | NI | single | 1 | 2 | 1 |

NI= No information.
